# A multi-dimensional risk signature for lupus nephritis in systemic lupus erythematosus: integrating symptoms, biochemistry and immune cell profiles

**DOI:** 10.3389/fimmu.2025.1680747

**Published:** 2025-11-07

**Authors:** Xiaoli Liu, Zhefeng Xiao, Xia Zhang, Jianfeng Li, Youhua Yuan, Na Li, Xiuzhi Zhang, Shiqing Li, Lan Gao

**Affiliations:** 1 Department of Clinical Laboratory, Henan Provincial People’s Hospital, People’s Hospital of Zhengzhou University, Zhengzhou, Henan, China; 2 Department of Pathology, NHC Key Laboratory of Cancer Proteomics, National Clinical Research Center for Geriatric Disorders, Xiangya Hospital, Central South University, Changsha, China; 3 Department of Pathology, Henan Medical College, Zhengzhou, China; 4 Henan Province Key Laboratory of Kidney Disease and Immunology, Henan Provincial People’s Hospital, People’s Hospital of Zhengzhou University, Zhengzhou, Henan, China; 5 Department of Ophthalmology, Henan Provincial People’s Hospital, People’s Hospital of Zhengzhou University, Zhengzhou, Henan, China

**Keywords:** lupus nephritis, systemic lupus erythematosus, PD-1, cystatin-C, T cell, risk model

## Abstract

**Background:**

Lupus nephritis (LN) is one of the most severe organ manifestations of systemic lupus erythematosus (SLE). Given its complex pathogenesis and heterogeneous clinical presentation, the clinical management of LN remains challenging. To identify risk factors for LN and provide new insights for its diagnosis and clinical treatment, it is essential to analyze the associations between demographic characteristics, biochemical parameters, clinical features, and immune cell profiles in SLE and LN.

**Methods:**

This retrospective study enrolled 121 SLE patients, including 55 with lupus nephritis (LN-positive) and 66 without LN (LN-negative), along with 121 age- and sex-matched healthy controls. Clinical manifestations and laboratory parameters were extracted from medical records for comparative analysis. Differences between groups were assessed using the Mann-Whitney *U* test and chi-square test. Spearman correlation analysis and regression modeling were employed to evaluate variable associations and their relationship with LN occurrence.

**Results:**

Compared to the LN-negative cases, LN patients were younger, had higher SLEDAI-2000 scores, ESR, WBC count, 24-hour urine total protein (24h-UTP), anti-dsDNA and ANA titers (AC-1 homogeneous pattern), and Cystatin-C (CysC), but lower C3 complement levels. They exhibited cutaneous manifestations and edema more frequently and arthritis less frequently. Flow cytometry showed higher circulating lymphocytes, CD3^+^CD8^+^ T cells, and PD-1^+^ T cell subsets (CD3^+^, CD4^+^, CD8^+^) in LN individuals. In LN patients, ESR correlated positively with PD-1^+^ T cell levels. In contrast, in LN-negative cases, anti-dsDNA levels correlated negatively with both age and PD-1^+^ T cell levels. Similarly, SLEDAI-2000 scores correlated negatively with lymphocytes and PD-1^+^CD3^+^ T cells. Multivariate regression analysis identified 24h-UTP, PD-1^+^CD4^+^ T cells, SLEDAI-2000 score, and edema as independent risk factors for LN in SLE.

**Conclusion:**

Significant differences were observed in both clinical manifestations and serological profiles between LN and LN-negative SLE patients. Notably, elevated PD-1^+^CD4^+^ T cells were identified as an independent risk factor for LN development. These findings suggest that abnormal expansion of PD-1^+^ T lymphocytes may serve as both a diagnostic marker for LN onset and a potential therapeutic target for LN management.

## 1 Introduction

Systemic lupus erythematosus (SLE) is an autoimmune disease characterized by loss of immune tolerance, autoantibody production, and immune-mediated damage to multiple organs.

Lupus nephritis (LN), a common and severe manifestation of SLE, is a major cause of both acute and chronic kidney injury and significantly contributes to morbidity and mortality in SLE patients (1, 2). Population-based studies report an overall annual incidence ranging from 1 to 8.7 cases per 100,000 person-years and a prevalence of 8 to 180 cases per 100,000 individuals (3, 4). Approximately 70–80% of SLE patients are at increased risk of developing LN, with about 10% progressing to end-stage renal disease (5, 6).

In some cases, LN may be clinically silent, with normal findings on urinalysis, renal function tests, and 24-hour urinary total protein (24h-UTP) excretion (7). When symptomatic, LN presents with renal dysfunction and urinary abnormalities, such as elevated cystatin C (CysC), hematuria, leukocyturia, cellular casts, and mild proteinuria, or with more sever clinical manifestations including nephrotic syndrome, acute nephritic syndrome, or rapidly progressive glomerulonephritis (8). LN also shares numerous clinical features overlap with non-renal SLE, including cutaneous/mucosal lesions, arthralgia/arthritis, edema, serositis, and multi-system involvement (gastrointestinal, respiratory, cardiovascular, or neurological). Disease activity is typically quantified using the SLEDAI-2000 score (9). The pathogenesis of LN involves multiple factors, including aberrant apoptosis, autoantibody generation, immune complex deposition, and complement activation. To identify risk factors for LN and predictive indicators of its development in SLE patients, a comprehensive investigation into the associations among clinical manifestations, serological markers, and immunological features is essential.

Standard laboratory monitoring includes erythrocyte sedimentation rate (ESR), autoantibody profiles, 24h-UTP, CysC, proteinuria, and complement component levels to evaluate treatment response and disease progression. However, the inherent heterogeneity of these biomarkers in SLE complicates both accurate disease assessment and the prediction of LN development (10). These challenges highlight the need for further investigation into the relationships between clinical manifestations and biomarkers in LN patients.

Immune dysfunction plays a central role in SLE pathogenesis, with T cells serving as key mediators through multiple mechanisms: facilitating B-cell activation, secreting pro-inflammatory cytokines and autoantibodies, infiltrating target tissues, and amplifying inflammatory responses-all contributing to multi-organ damage (11). However, dysregulation of co-inhibitory checkpoints may disrupt T-cell exhaustion mechanisms, potentially promoting autoimmunity (12). Among these checkpoints, programmed death-1 (PD-1) is particularly crucial. The PD-1 signaling pathway is essential for maintaining immune tolerance, as evidenced by studies showing that PD-1 deficiency or blockade accelerates disease progression in various autoimmune mouse models (13–15).

Previous studies have identified expanded populations of PD-1^+^CD4^+^ T cells in SLE patients, with their frequency positively correlating with disease activity (16, 17). CD8^+^ T cells have also been implicated in LN pathogenesis through renal infiltration and association with disease severity (18). Notably, a distinct hyperactivated PD-1^+^CD8^+^ T-cell subset has been characterized in LN patients (19). These findings highlight the need for comprehensive quantification of circulating lymphocytes and T-cell subsets in SLE patients, along with systematic evaluation of their association with LN development. In the current study, we examined the interrelationships between clinical manifestations, biochemical markers, disease activity (SLEDAI-2000), and immune cell profiles in SLE patients. Furthermore, we developed a predictive risk model for LN to advance diagnostic approaches and inform clinical management strategies.

## 2 Materials and methods

### 2.1 Data collection and processing

**Figure 1:**
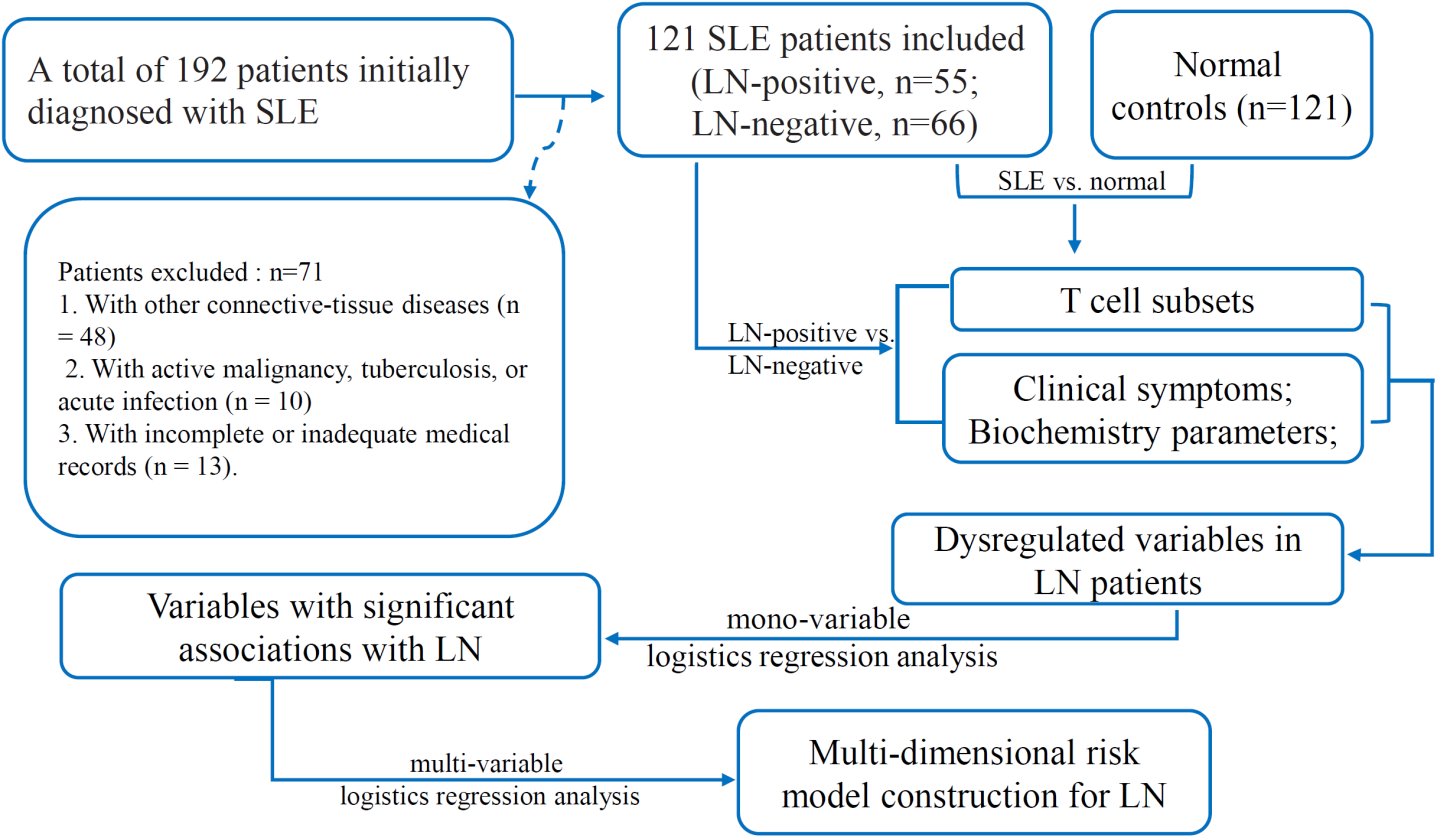
The flow diagram of the screening and enrollment of the patients in this study.

This retrospective study was conducted at Henan Provincial People’s Hospital (Zhengzhou, China). A total of 192 patients with a confirmed diagnosis of SLE who had been admitted to either the Department of Rheumatology and Immunology or the Department of Nephrology between January 2023 and December 2024 were enrolled. Patients who met either the 2019 EULAR/ACR classification criteria for SLE (20) or the 2012 SLICC criteria (21) and who had only received corticosteroid therapy at the time of data collection were included. This uniform treatment approach was adopted to minimize the potential confounding effects of different treatment regimens on the biochemical and immunological parameters assessed in our study. The following exclusion criteria were applied (1): coexistence of other connective-tissue diseases, such as rheumatoid arthritis, primary Sjögren’s syndrome, mixed connective-tissue disease, vasculitis, polymyositis, or dermatomyositis (n = 48); (2): active malignancy, tuberculosis, or acute infection (n = 10); and (3) incomplete or inadequate medical records (n = 13). Consequently, 71 patients were excluded, leaving a total of 121 SLE patients who met the inclusion criteria and were included in the final analysis (**Figure 1**). Of these, 55 had biopsy-confirmed LN (LN-positive, LN-posi group) and 66 did not (LN-negative, LN-neg group). Renal biopsies were performed using standard techniques, and the biopsy specimens were processed for light microscopy, immunofluorescence, and electron microscopy (20). The renal biopsy findings were classified according to the International Society of Nephrology/Renal Pathology Society (ISN/RPS) classification system for LN (21). Among the LN patients, the most common histological class was class IV (diffuse proliferative glomerulonephritis), identified in 20 cases (36.36%). This was followed by class III+V (n = 9, 16.36%), class V (n = 7, 12.73%), class III (n = 6, 10.91%), and class III+IV (n = 6, 10.91%), respectively. Class II was identified in 4 cases (7.27%), and class IV+V was detected in only 3 patients (5.45%). Interestingly, histological classes I and VI were not found in any of the 55 LN patients. The demographic characteristics, clinical manifestations, laboratory parameters, and LN classification were extracted from the electronic medical record system. The clinical and demographic characteristics of SLE patients, as well as the controls, are outlined in **Supplementary Table S1** and are visualized in **Supplementary Figure S1**. For each patient, we collected the most recent clinical and laboratory test results obtained within one week prior to renal biopsy to ensure that the biochemical indicators accurately reflected the patients’ current disease status. An age- and sex-matched control group of 121 healthy individuals (normal group) was recruited from the physical-examination center. Disease activity in the SLE cohort was quantified using the SLE Disease Activity Index 2000 (SLEDAI-2K) (9). Blood samples for cytometer analysis were collected as part of routine clinical care for SLE patients and during routine check-ups for healthy age- and sex-matched controls, and were anonymized and stored in the hospital’s biobank for research purposes. The study was approved by the Ethics Committee of Henan Provincial People’s Hospital (Approval Number: 2024-96). The Ethics Committee waived the requirement for informed consent due to the retrospective nature of the study and the use of anonymized data.

### 2.2 The clinical symptoms and the serological biomarkers

Clinical characteristics—including fever, cutaneous involvement, arthralgia/arthritis, serositis, edema, hematological abnormalities, neuropsychiatric manifestations, and gastrointestinal involvement—were extracted from patients’ medical records. Anti-dsDNA antibodies were quantified by ELISA (Euroimmun AG, Lübeck, Germany). Antinuclear antibodies (ANAs) were detected by indirect immunofluorescence (IIF) on HEp-20-10/liver biochip slides (Euroimmun AG, Lübeck, Germany). A serum sample was considered ANA-positive when characteristic IIF staining was observed at a 1:100 dilution. ANA level was graded semi-quantitatively (1+ to 4+) when positive at dilutions of 1:100, 1:320, 1:1000, and 1:3000. IIF pattern interpretation followed the international consensus recommendations for clinical ANA testing (22). Extractable Nuclear Antigen (ENA) test panels were used to detect a more specific set of autoantibodies, including anti-U1-nRNP/Sm, anti-Sm, anti-Ro52, anti-SSA, anti-SSB anti-nucleosome (AnuA), anti-histone (AHA) and anti-ribosomal (anti-P), which were determined by line-blot immunoassay (EUROLINE kits; Euroimmun AG, Lübeck, Germany). Serum immunoglobulins (IgG, IgM, IgA) and complement components (C3, C4) were quantified by immunoturbidimetry on a Roche Cobas 6000 E501 analyzer. Complete blood counts (white blood cells, red blood cells, platelets and hemoglobin) were acquired using an automated hematology analyzer (Automatic Hematology Analyzer XR™, Sysmex Co.). 24h-UTP and CysC were measured on an ARCHITECT c8000 analyzer by pyrogallol red–molybdate and turbidimetric inhibition immunoassay, respectively. ESR was determined with an automatic ESR analyzer (TEST1/THL; ALIFAX).

### 2.3 Flow cytometry analysis

Lymphocytes and T-cell subsets (CD3^+^ T cells, CD3^+^CD4^+^ T cells, CD3^+^CD8^+^ T cells, CD3^+^PD-1^+^ T cells, CD4^+^PD-1^+^ T cells and CD8^+^PD-1^+^ T cells) were enumerated by flow cytometry (Wmini5268; Guangzhou Weimi Bio-Tech Co., Ltd.) according to the manufacturer’s instructions. We employed a sequential gating strategy to accurately identify and analyze the lymphocytes and T-cell subsets of interest. The gating strategy was designed to ensure the precise identification of specific cell subsets based on their phenotypic markers. Initially, we gated on live cells using forward scatter (FSC) and side scatter (SSC) parameters to exclude debris and dead cells. Subsequently, we identified the lymphocyte cluster based on CD45 expression (using an APC-Cy7-conjugated anti-CD45 antibody) and SSC parameters. Within the lymphocyte gate, CD3^+^ T cells were identified using CD3 staining, and further subsets were delineated by gating on CD3^+^CD4^+^ T cells and CD3^+^CD8^+^ T cells using CD4 and CD8 staining, respectively. Additionally, we identified PD-1 positive T-cell subsets within the CD3^+^ T cells, specifically gating on CD3^+^PD-1^+^ T cells, CD3^+^CD4^+^PD-1^+^ T cells, and CD3^+^CD8^+^PD-1^+^ T cells using PD-1 staining. To ensure the consistency and accuracy of our gating strategy, we performed quality control checks using isotype controls and fluorescence minus one (FMO) control, which helped us to accurately set the gates and avoid any false-positive or false-negative results. A panel of antibodies to label different cell surface markers was used in the cytometer analysis. The antibodies used included anti-CD45 (APC-Cy7, catalog number: 3060010025), anti-CD3 (FITC, catalog number: 3060030063), anti-CD4 (APC, catalog number: 3060010020), anti-CD8 (PerCP, catalog number: 3060010120), and anti-PD-1 (PE, catalog number: 3060010015). These antibodies were sourced from Weimi Bio-Tech Co., Ltd. T-cell subset distributions in patients and controls were compared, and the clinical characteristics, serum biomarkers, autoantibody profiles and T-cell subsets of the patients were analyzed according to LN status (LN-posi versus LN-neg).

### 2.4 Statistical analysis

R 4.3.1 software was used for data analyses and visualization. Mann-Whitney U was used for comparisons of the quantitative variables. Chi-square test was used for the comparisons of the qualitative variables. Spearman correlation analysis was used to investigate the potential associations between the levels of the variables. For all the analyses, *p* < 0.05 was considered significant. Univariate logistic regression analysis was conducted on the biomarkers and clinical symbols that showed significant differences between the LN-positive and LN-negative groups to explore their associations with LN occurrence. Then, the variables with *p* < 0.001 were applied to multi-variable logistics regression analysis to construct a risk model for LN occurrence. A nomogram was created by the results of the logistics regression model to evaluate and visualize the probability of the cases to be LN.

## 3 Results

### 3.1 The differences of age, clinical symptoms and autoantibody levels between LN-positive and LN-negative groups

**Figure 2:**
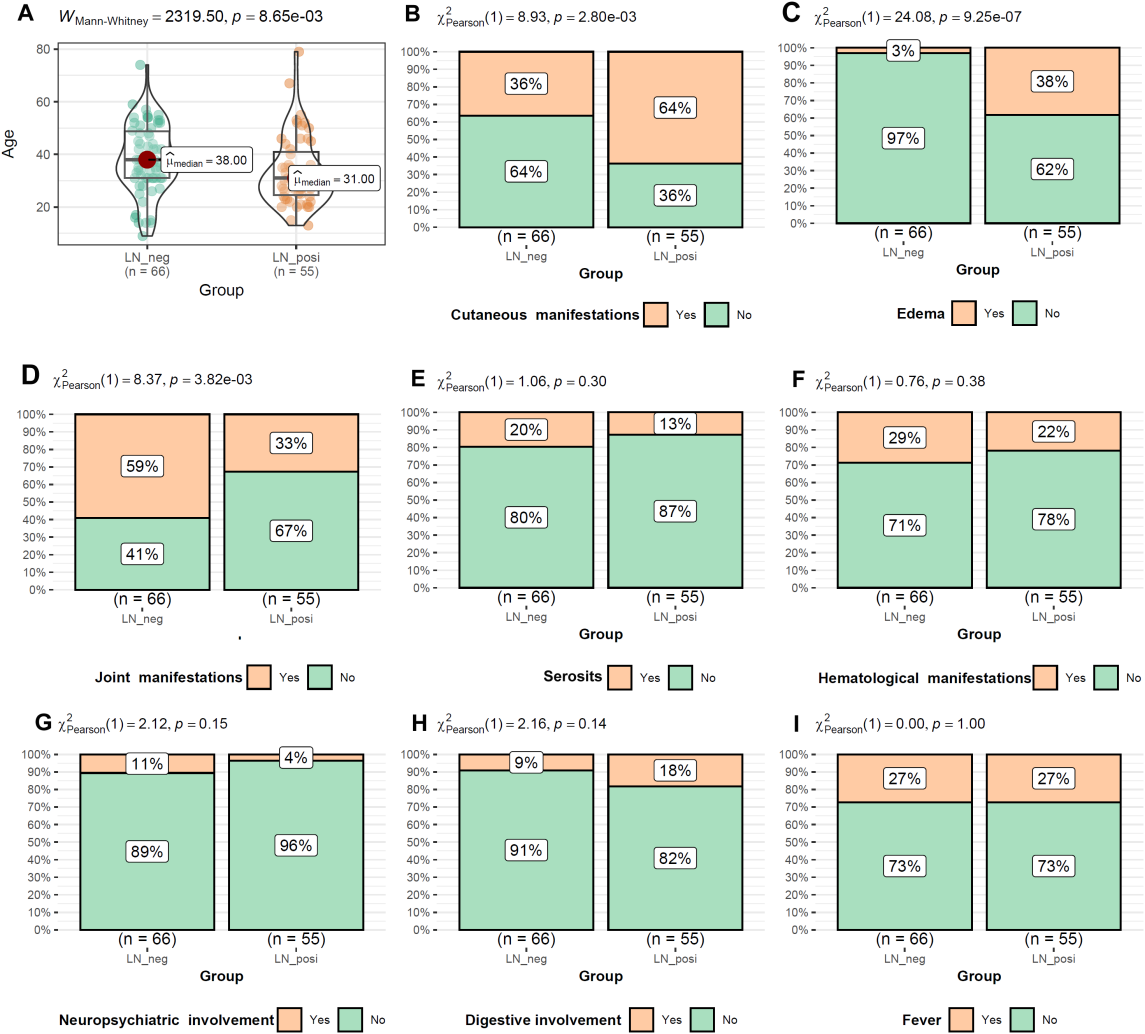
The comparisons of age and the symptom occurrences between LN-positive and LN-negative groups **(A–I)**. Mann-Whitney U test and Chi-square test were used for comparisons and *p* < 0.05 was considered significant.

**Figure 3:**
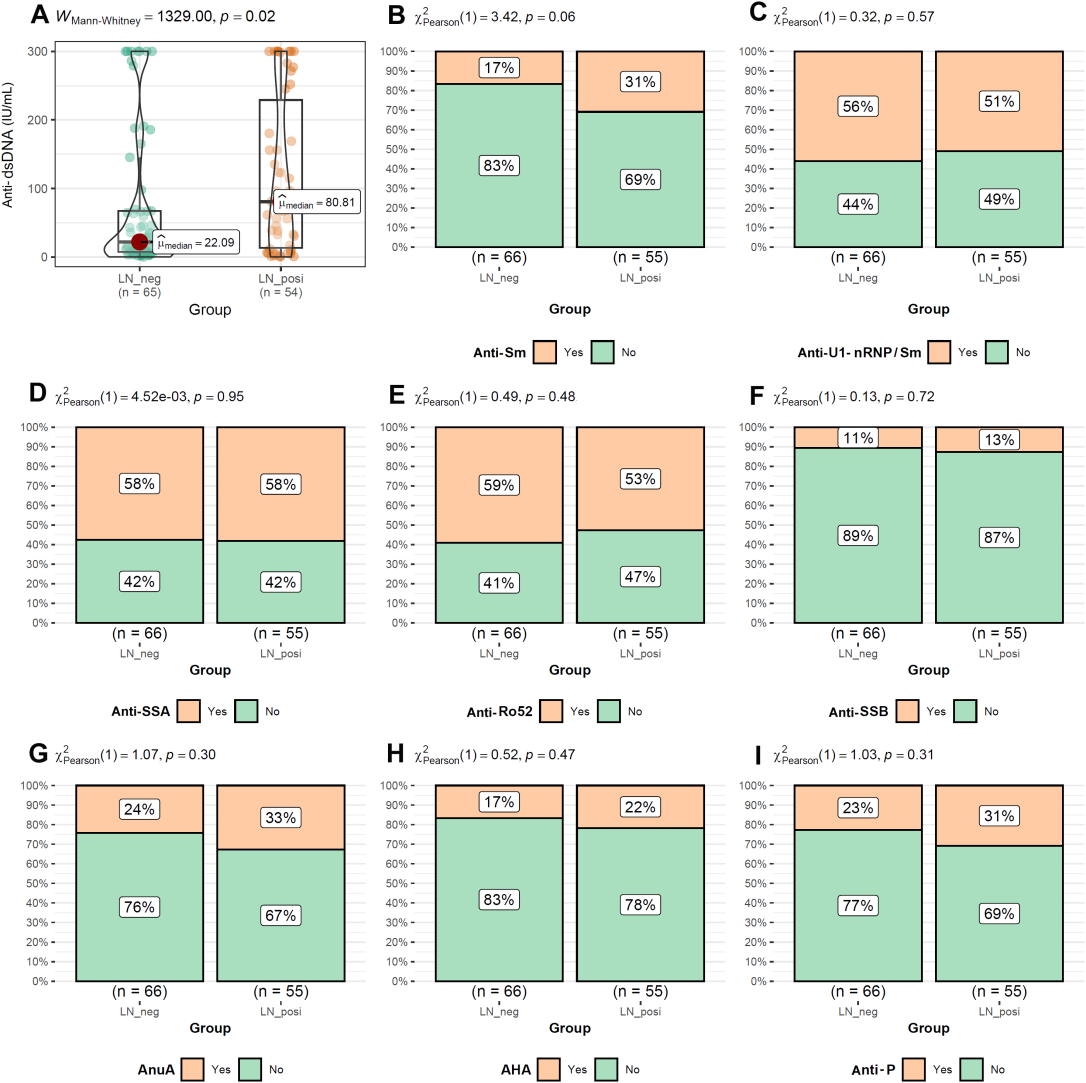
The comparisons of autoantibody levels between LN-positive group and LN-negative group. **(A)** Higher level of Anti-dsDNA in LN patients than LN-negative cases. **(B–I)** Comparable levels of Anti-Sm, Anti-U1-nRNP/Sm, Anti-SSA, Anti-Ro52, Anti-SSB, AnuA, AHA and anti-P between LN patients than LN-negative cases. The Mann-Whitney U test was used to analyze quantitative data, while the Chi-square test was used to analyze qualitative data. *P* < 0.05 was considered to be statistically significant.

**Figure 4:**
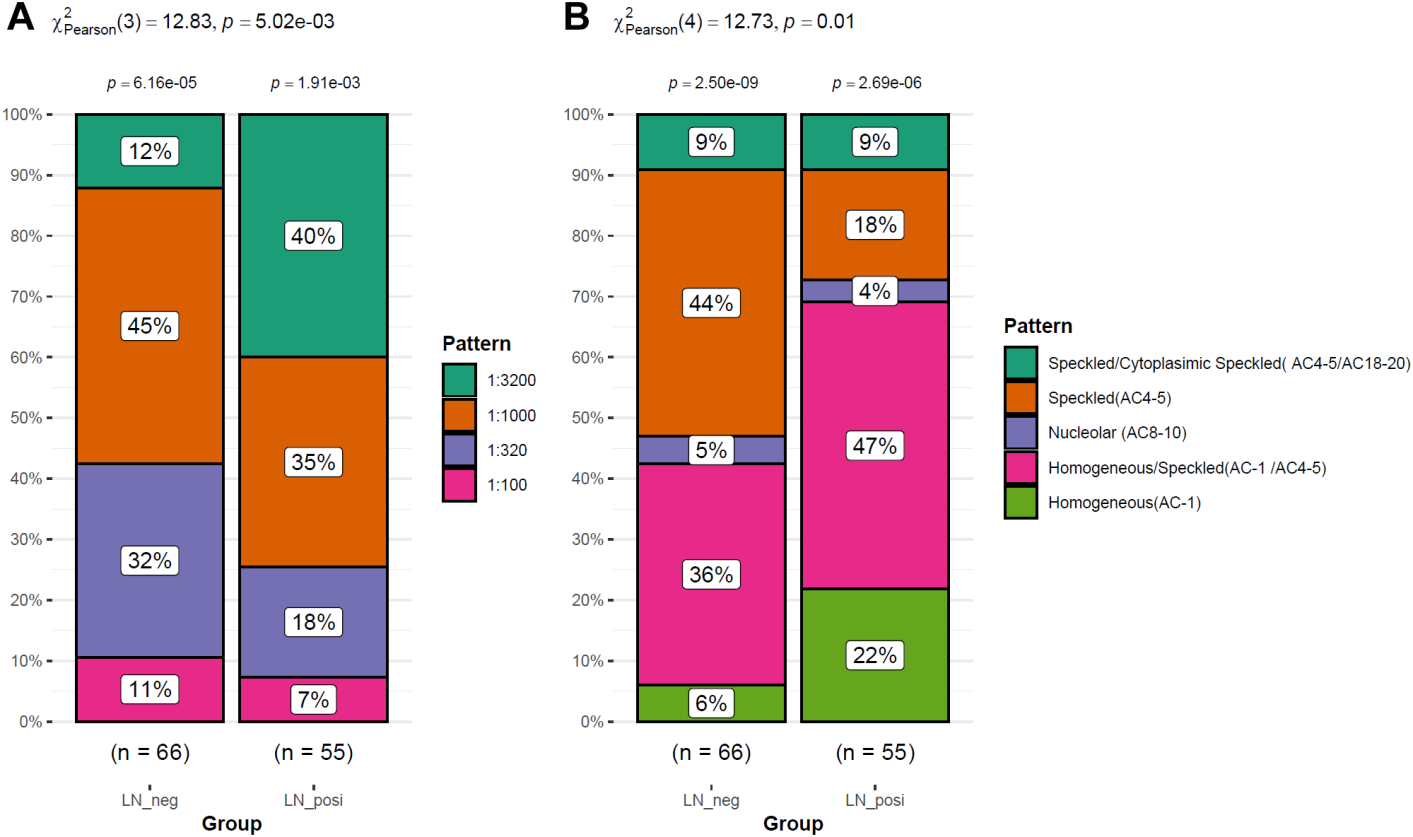
The ANA differences between LN patients and LN-negative cases. **(A)** Different distribution of ANA levels between LN-positive and LN-negative cases. **(B)** The difference of the staining patterns of ANA between LN patients and LN-negative cases.

As shown in **Figure 2A**, the LN patients were younger than that of LN-negative cases. For the symptoms, the incidences of cutaneous manifestations and edema were significantly higher in LN patients than LN-negative cases (**Figures 2B, C**). However, the incidence of joint manifestations was significantly lower in LN patients than LN-negative cases (**Figure 2D**). While, no significant difference of other clinical symptoms was shown between the two groups (**Figures 2E–I**). For the autoantibodies, anti-dsDNA level was shown to be higher in LN patients than LN-negative cases (**Figure 3A**) while no significant difference of the positive rate of ENA (anti-U1-nRNP/Sm, anti-Sm, anti-Ro52, anti-SSA, anti-SSB, anti-AnuA, anti-AHA, anti-P) were shown (**Figures 3B–I**). For ANA titers, the LN-positive group showed a markedly higher proportion (40%) of sera with a 1:3200 dilution than the LN-negative group (**Figure 4A**). Within the LN-positive cohort, 75% of patients exhibited ANA titers above 1:1000. Conversely, LN-negative cases predominantly displayed moderate to strong titers, ranging from 1:320 to 1:1000 (**Figure 4A**). These findings indicate a strong association between elevated ANA titers and LN. Moreover, the distribution of ANA staining patterns differed between the two groups. In the LN cohort, the combined homogeneous/speckled pattern (AC-1/AC-4-5) predominated, whereas the purely homogeneous pattern (AC-1) was significantly more frequent in LN patients than in LN-negative cases (**Figure 4B**).

### 3.2 The differences of SLE activity index, blood cell counts and immune-related molecules between LN patients and LN-negative cases

**Figure 5:**
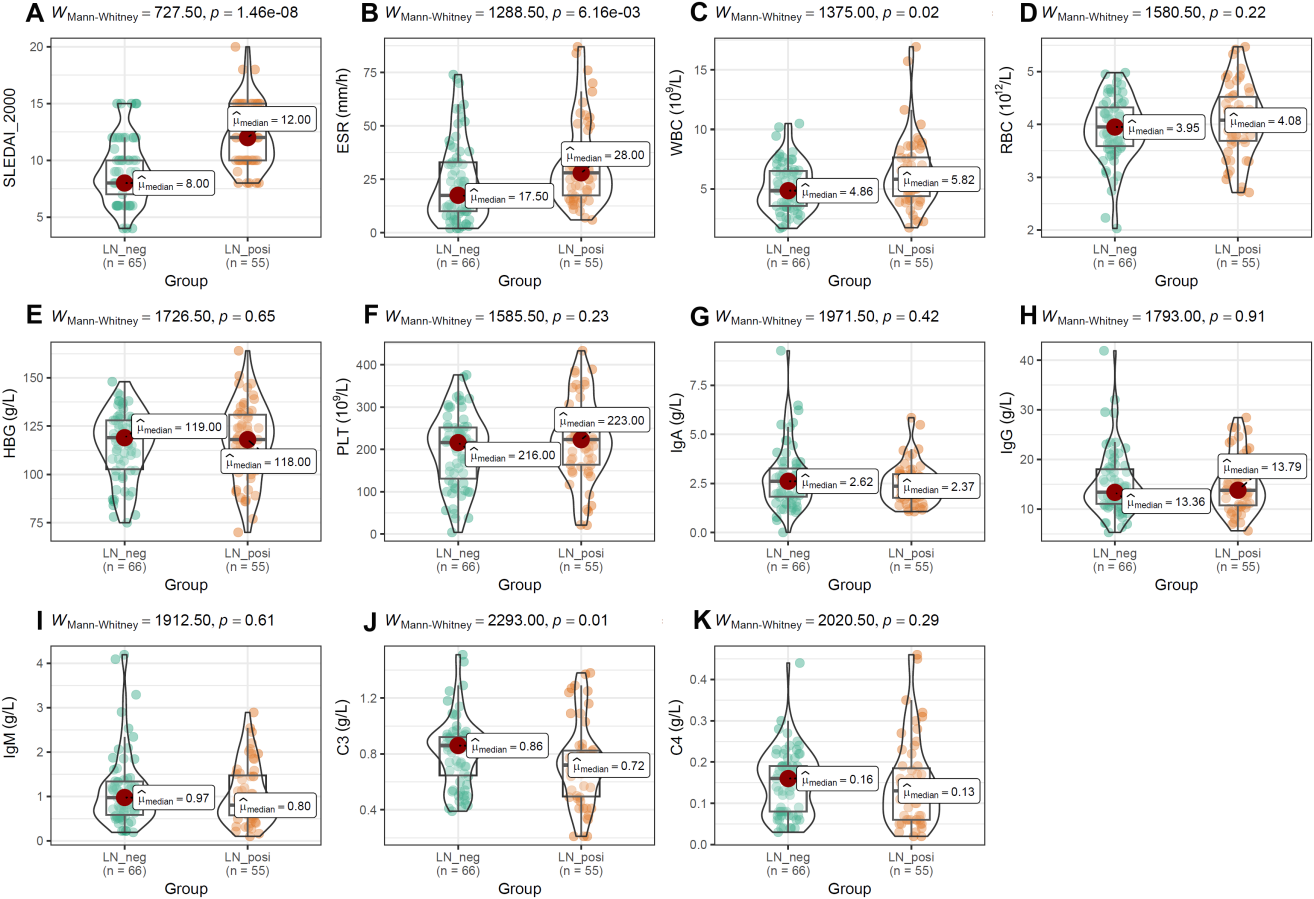
The comparisons of activity index, blood cell counts and immune-related molecules between the two groups. **(A, B)** The difference of SLEDAI-2000 and ESR between LN-positive and LN-negative cases. **(C–I)** The comparisons of blood cell counts, HBG and immunoglobulin (IgA, IgG, IgM) between LN patients and LN-negative cases. **(J, K)** The comparisons of complement (C3, C4) between LN-positive group and LN-negative group. The Mann-Whitney U test was used for analysis and *p*-value< 0.05 was considered to be statistically significant.

As shown in **Figure 5**, SLEDAI-2000 and ESR levels were significantly higher in LN patients than in LN-negative cases (**Figures 5A, B**). Among blood-cell indices, only WBC differed significantly between the two groups (**Figure 5C**), whereas RBC, hemoglobin and platelet counts did not (**Figures 5D–F**). Serum immunoglobulins (IgA, IgG, IgM; **Figures 5G–I**) and complement C4 (**Figure 5K**) were comparable between groups. In contrast, complement C3 was significantly lower in LN patients than in LN-negative subjects (**Figure 5J**).

### 3.3 Differential distribution of T-cell subsets and difference of renal function in SLE patients with and without LN

**Figure 6:**
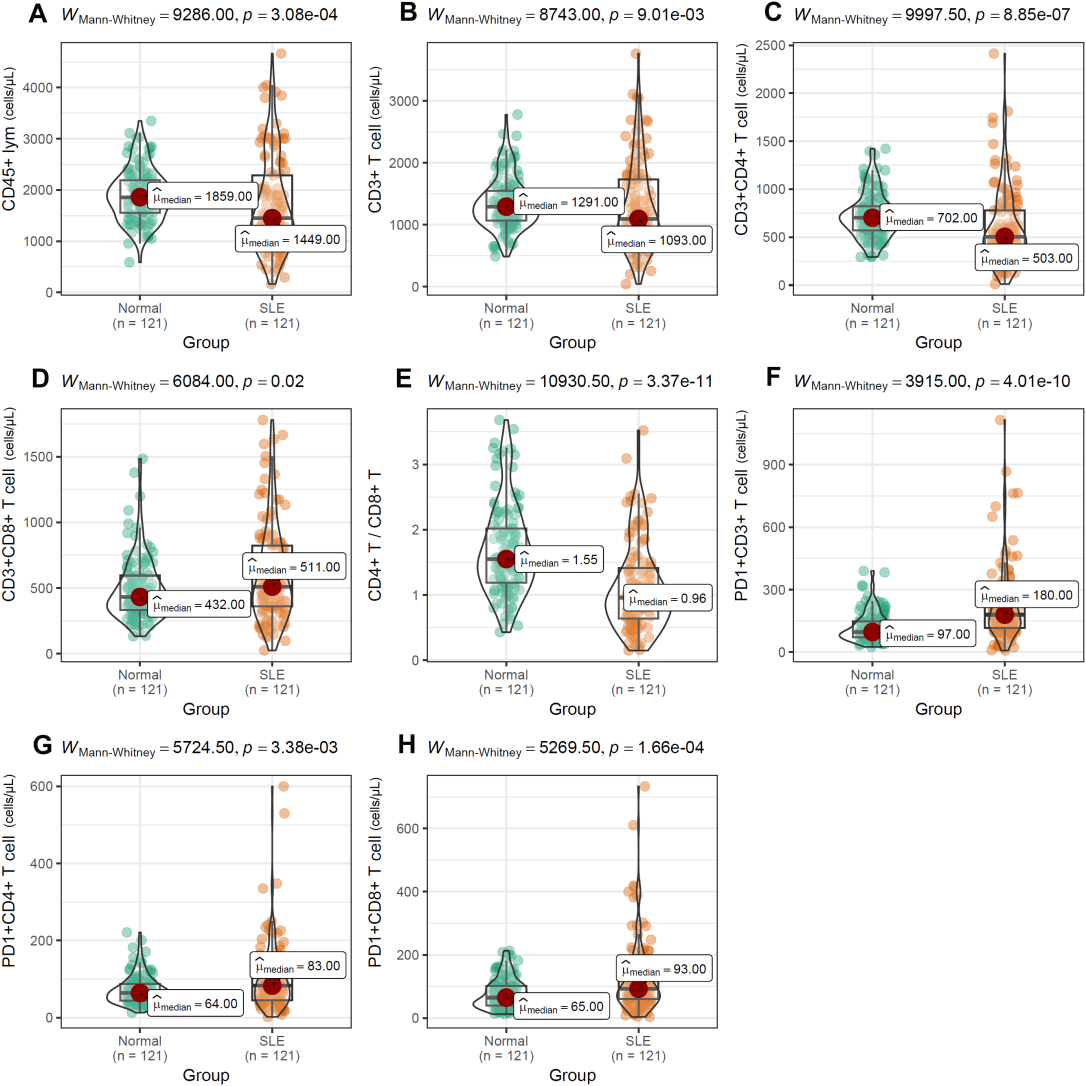
Differential distribution of T-cell subsets **(A–H)** between SLE patients and normal controls. Mann-Whitney U test was used and *p* value< 0.05 was considered to be statistically significant.

**Figure 7:**
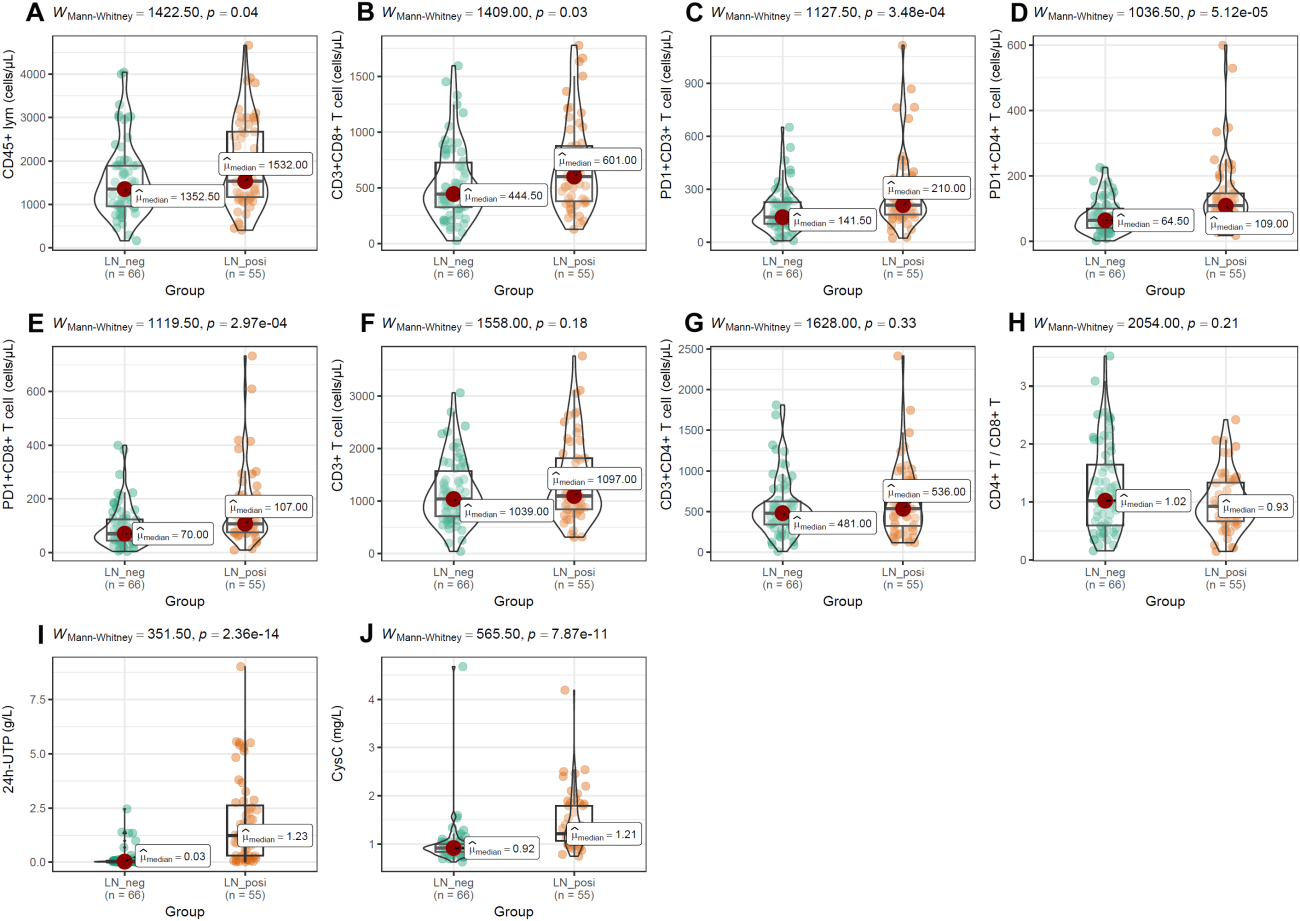
Differences of T-cell subset distributions and renal function indices between LN patients and LN-negative cases. **(A–H)** The comparisons of T-cell subset distributions between SLE patients with and without LN. **(I, J)** Significant difference of 24h-UTP and CysC between LN patients and LN-negative cases. Mann-Whitney U test was used and *p* < 0.05 was considered to be statistically significant.

To evaluate the impact of PD-1 on distinct T-cell compartments, we enumerated these subsets in peripheral-blood mononuclear cells (PBMCs) by multiparameter flow cytometry. As shown in **Figure 6**, absolute counts of CD45^+^ lymphocytes (**Figure 6A**), CD3^+^ T cells (**Figure 6B**), CD3^+^CD4^+^ T cells (**Figure 6C**), and the CD4^+^/CD8^+^ T-cell ratio (**Figure 6E**) were all significantly lower in SLE patients than in normal controls (NCs). In contrast, the frequencies of CD3^+^CD8^+^ T cells (**Figure 6D**), PD-1^+^CD3^+^ T cells (**Figure 6F**), PD-1^+^CD4^+^ T cells (**Figure 6G**), and PD-1^+^CD8^+^ T cells (**Figure 6H**) were markedly elevated in SLE patients compared with NCs. Among SLE patients, LN individuals exhibited significantly higher frequencies of CD45^+^ lymphocytes, CD8^+^ T cells, PD-1^+^CD3^+^ T cells, PD-1^+^CD4^+^ T cells, and PD-1^+^CD8^+^ T cells than LN-negative cases (**Figures 7A–E**). However, absolute counts of CD3^+^ T cells, CD4^+^ T cells, and the CD4^+^/CD8^+^ T-cell ratio did not differ significantly between the two SLE subgroups (**Figures 7F–H**). Consistent with renal impairment, both 24h-UTP and serum CysC were significantly elevated in the LN-positive group relative to the LN-negative group (**Figures 7I, J**).

### 3.4 The heterogeneity of the correlations between the serum indices with T cell subpopulation levels in SLE patients

**Figure 8:**
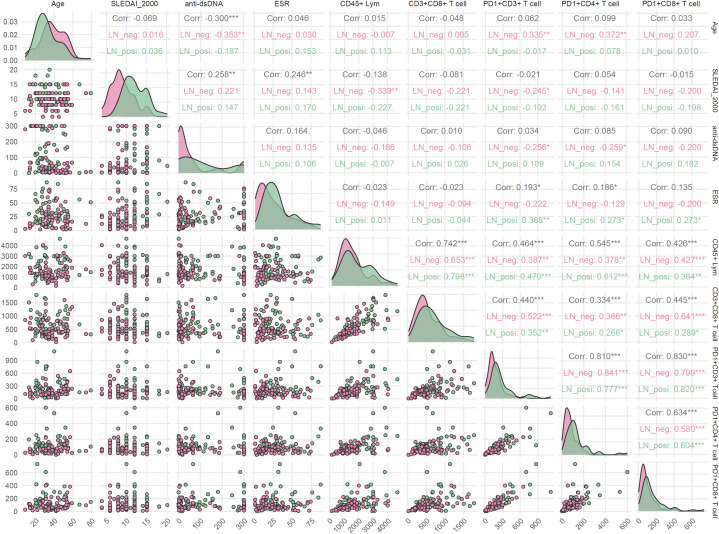
Correlations between clinical parameters and T-cell subsets in LN-positive and LN-negative SLE patients. Spearman correlation was used and *p* < 0.05 was considered statistically significant.

Because age, ESR, SLEDAI-2000, and anti-dsDNA levels differed significantly between LN and LN-negative cases, their associations with T-cell subsets were evaluated. As illustrated in **Figure 8**, the two groups exhibited distinct correlation patterns. In LN patients, ESR correlated positively with the frequencies of PD-1^+^CD3^+^ T cells (*r* = 0.388, *p* < 0.01), PD-1^+^CD4^+^ T cells (*r* = 0.273, *p* < 0.05), and PD-1^+^CD8^+^ T cells (*r* = 0.273, *p* < 0.05), whereas these associations were absent in LN-negative cases. Conversely, age showed positive correlations with PD-1^+^CD3^+^ T cells (*r* = 0.335, *p* < 0.01) and PD-1^+^CD4^+^ T cells (*r* = 0.372, *p* < 0.01) only in LN-negative cases. Similarly, anti-dsDNA antibody levels were negatively associated with age (*r* = –0.353, *p* < 0.01), PD-1^+^CD3^+^ T cells (*r* = – 0.256, *p* < 0.05), and PD-1^+^CD4^+^ T cells (*r* = – 0.259, *p* < 0.05) exclusively in the LN-negative group. This heterogeneity points to distinct immune-regulatory mechanisms in LN patients compared with their LN-negative counterparts. In addition, several correlations were shared between the two groups. The positive intercorrelations observed among T-cell subsets are consistent with their common ontogeny and coordinate regulation during immune activation.

### 3.5 Risk model construction for LN patients

**Figure 9:**
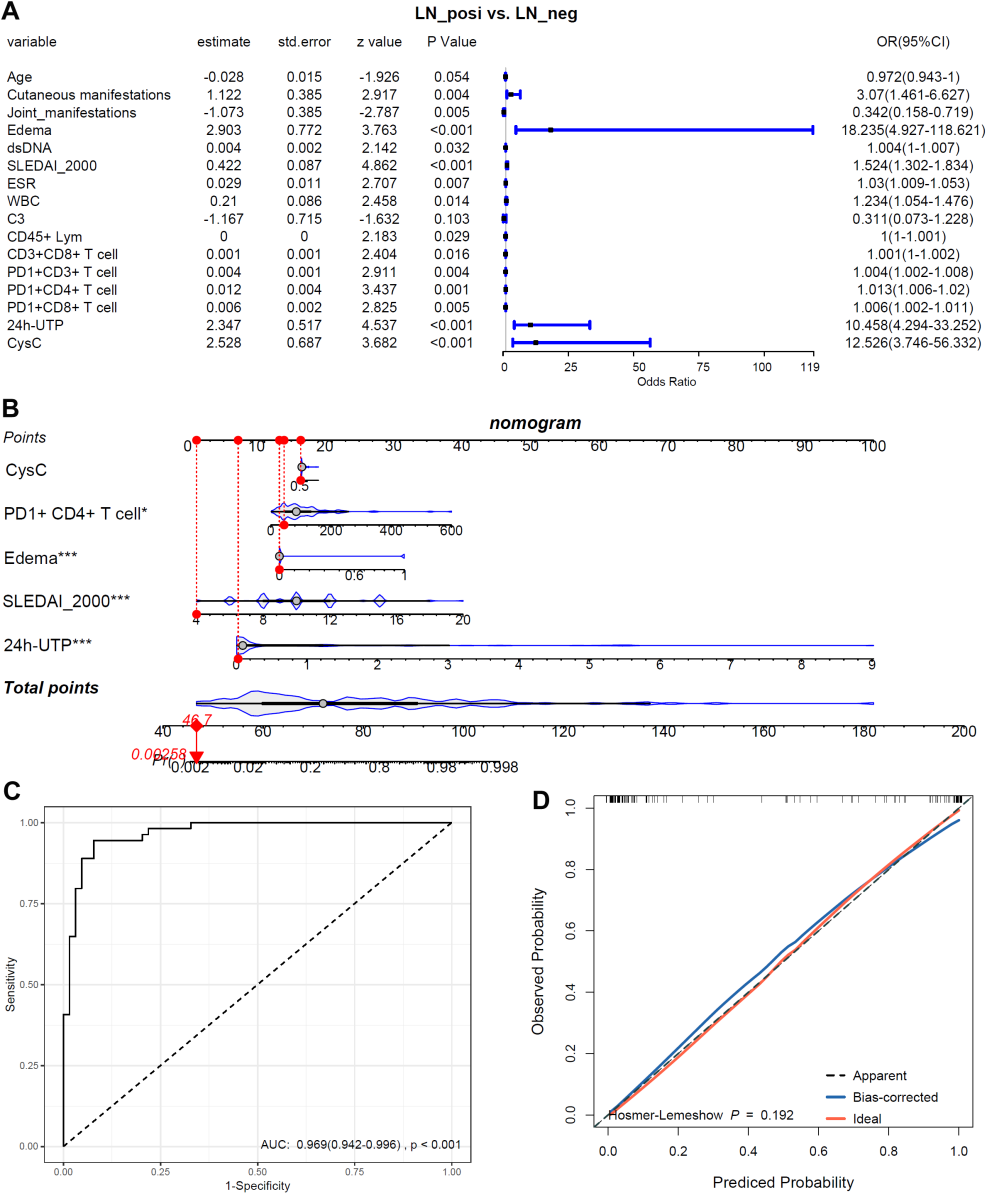
The associations of the biomarkers and symbols with LN-positive status. **(A)** Mono-variable logistic regression analysis of the biomarkers and symbols in LN patients. **(B)** Multi-variable logistics regression analysis of the biomarkers and symbols in LN patients. **(C)** The ROC of the risk model in discriminating LN patients from LN-negative cases. **(D)** The calibration curve of the risk model. *, p<0.05; ***, p<0.001.

To identify risk factors for LN, all variables that differed significantly between LN and LN-negative cases (*p* < 0.05) were entered into a univariate logistic regression analysis. These variables included laboratory indices (SLEDAI-2000, anti-dsDNA, ESR, WBC, CD45^+^ lymphocytes, CD3^+^CD8^+^ T cells, PD-1^+^CD3^+^ T cells, PD-1^+^CD4^+^ T cells, PD-1^+^CD8^+^ T cells, 24h-UTP, and CysC) and clinical manifestations (cutaneous involvement, joint involvement, and edema). As shown in **Figure 9A**, all examined variables except age and C3 were significantly associated with the presence of LN. Joint manifestations appeared to be protective (OR < 1, *p* < 0.05), whereas elevated SLEDAI-2000, anti-dsDNA antibodies, ESR, WBC, CD45^+^ lymphocytes, CD3^+^CD8^+^ T cells, PD-1^+^CD3^+^ T cells, PD-1^+^CD4^+^ T cells, PD-1^+^CD8^+^ T cells, 24h-UTP, CysC, as well as cutaneous involvement and edema, were all identified as risk factors for LN in SLE patients (OR > 1, *p* < 0.05). The five variables (CysC, 24h-UTP, PD1^+^CD4^+^T, SLEDAI-2000 and edema) with *p* ≤ 0.001 in the mono-variable regression analysis were applied to multi-variable analysis to identify independent risk factors for SLE with LN. As shown in **Figure 9B**, 24h-UTP, PD1^+^CD4^+^T, SLEDAI-2000 and edema were indicated to be independent risk factors for SLE with LN. As shown in **Figure 9C**, the risk model could discriminate LN patients from LN-negative cases effectively, with an AUC of 0.969 (95%CI: 0.942-0.996). As shown by the calibration curve (**Figure 9D**), the model’s predicted probabilities are highly reliable (*p* = 0.192, *p* > 0.05).

## 4 Discussion

LN, in some cases, serves as the initial clinical manifestation leading to a SLE diagnosis (23), while so-called “silent” LN—characterized by normal urinalysis, preserved renal function, and absence of proteinuria in asymptomatic individuals—is frequently observed (24). Despite substantial progress in elucidating the genetic and pathogenic mechanisms of LN over the past few decades, which has led to the refinement of therapeutic strategies, accurately assessing disease activity and reliably predicting the onset of LN remain significant clinical challenges. To address this unmet need, we developed an integrated risk model combining clinical parameters with biological markers. Our analysis identified several independent predictors of LN, providing novel evidence to guide clinical management and improve long-term prognostication.

The clinical characteristics of LN patients are highly heterogeneous (25). In this study, LN patients frequently exhibited skin and mucosal damage, as well as edema (e.g., lower limbs, eyelids, and face). In contrast, joint symptoms were more common in LN-negative cases. Previous research (26–28) has documented a higher prevalence of LN in juvenile-onset SLE compared to adult-onset SLE. In line with these findings, our study revealed that LN patients were significantly younger than LN-negative cases. This dichotomy suggests that LN may represent a distinct immunologic endotype associated with a more aggressive disease course. Conversely, the joint-predominant pattern observed in LN-negative SLE may reflect a milder and more limited disease spectrum.

ANA serve as a key immunologic hallmark of systemic SLE, with distinct ANA staining patterns reflecting antibodies targeting different antigens (29, 30). Previous studies have reported associations between various SLE subtypes and specific IIF-ANA staining patterns (31). In the present study, we also observed distinct ANA patterns in the two groups. Notably, double-stranded DNA (dsDNA), a primary target antigen for ANA (AC-1), has been previously linked to renal involvement (32). Supporting these observations, our study found significantly higher anti-dsDNA levels in LN patients compared to LN-negative cases.

Several T cell subset abnormalities have been identified in SLE patients and are implicated in disease immunopathogenesis (33). In our study, the LN-positive group exhibited more pronounced T cell dysregulations. Elevated activated CD8^+^ T cells have been associated with increased disease activity and renal involvement (34). However, we observed higher total CD8^+^ T cell counts in LN patients compared to LN-negative cases. Notably, we found no significant correlation between total CD8^+^ T cell counts and SLEDAI-2000 scores in our LN cohort. This may be because our analysis measured total CD8^+^ T cells rather than specifically assessing the activated subset.

PD-1, a key immune checkpoint molecule in LN, has a dual function: it can both suppress immunity and, paradoxically, promote inflammation (19, 35, 36). Our study found significant increases in PD-1^+^CD3^+^T, PD-1^+^CD4^+^, and PD-1^+^CD8^+^ T cell populations in LN patients. Importantly, the frequency of these subsets correlated positively with ESR levels, suggesting a link to systemic inflammation. This indicates that PD-1^+^ T cells in LN may not just be exhausted or regulatory but actively contribute to inflammation and renal damage. Moreover, the different correlations between PD-1^+^ T cell frequencies and clinical parameters in LN versus LN-negative patients underscore the context-specific nature of PD-1’s immune regulation, highlighting that its pathogenic role is shaped by the unique immunological environment of active LN.

As the most severe complication of SLE, LN has an unpredictable onset and disease course. A reliable risk prediction model is urgently needed to identify high-risk patients early, guide individualized monitoring, and prevent irreversible renal damage. To address this, we developed a nomogram-based LN risk prediction model. Nomograms are visual tools that estimate the probability of clinical outcomes by integrating multiple predictive factors (37). Such models can enhance stratification and support clinical decision-making by accounting for patient-specific variables.

Our study identified 24h-UTP, PD-1^+^CD4^+^ T cells, SLEDAI-2000 score, and edema as independent risk factors for LN. PD-1^+^CD4^+^ T cells, which infiltrate renal tissue and release pro-inflammatory cytokines such as IFN-γ, act as key drivers of inflammation (38). Elevated 24h-UTP not only predicts renal flare and long-term outcomes (39, 40) but also contributes mechanistically to edema formation, highlighting its dual role as a prognostic and pathophysiologic marker (41). Similarly, higher SLEDAI-2000 scores reflect active LN and inform treatment intensification (42). By integrating these parameters, our model provides a clinically actionable framework for personalized monitoring and timely intervention to mitigate irreversible kidney damage. This approach could improve patient outcomes by facilitating early identification and management of high-risk patients.

Although this study presents an integrated risk model for LN combining clinical, biochemical, and immunological parameters, several limitations should be noted. First, the sample size was relatively small, and the cohort was restricted to Central China; we are now expanding recruitment to a multi-province consortium to test the model’s generalizability. Second, although we examined PD-1^+^ T-cell distributions, further studies—such as flow-sorted subset analysis and single-cell RNA sequencing—are needed to clarify their functional phenotypes and signaling pathways. Finally, we did not assess other immune checkpoint markers (e.g., CTLA-4, LAG-3) or additional T cell activation markers. Future research should include a broader panel and deeper investigation of multiple immune checkpoint markers to better understand the roles of T cells in LN. These studies will improve the model’s diagnostic accuracy and help identify new therapeutic targets for LN.

## 5 Conclusion

In this retrospective, multi-dimensional study, we systematically characterized the clinical, biochemical, and immunological differences between SLE patients with and without LN. By integrating these diverse datasets, we developed a robust LN risk prediction model that demonstrates excellent calibration and discriminative performance. The model not only quantifies individual probabilities of LN occurrence but also identifies key pathogenic mechanisms that could be targeted therapeutically. Collectively, our findings provide new insights into T-cell-mediated immune dysregulation in LN pathogenesis and offer clinicians an evidence-based tool for early detection, risk stratification, and personalized management of LN.

## Data Availability

The original contributions presented in the study are included in the article/**Supplementary Material**. Further inquiries can be directed to the corresponding authors.

## Supplementary material

Supplementary Table 1 Clinical, demographic, autoantibodies characteristics and historical classifications of SLE patients.

Supplementary Figure 1 Heatmap of demographic, clinical, biochemical, immunological, and histological features in LN patients.
