# Supplementary material for: A multi-dimensional risk signature for lupus nephritis in systemic lupus erythematosus: integrating symptoms, biochemistry and immune cell profiles
Source: Front Immunol. 2025 Nov 7;16:1680747. doi: 10.3389/fimmu.2025.1680747 (PMC12634612; doi:10.3389/fimmu.2025.1680747)
Supplement: Supplementary Table 1 — Clinical, demographic, autoantibodies characteristics and historical classifications of SLE patients. [file DataSheet1.pdf]

Table S1. Clinical, demographic, autoantibodies characteristics and historical classifications of SLE patients

	**SLE patients (n=121)**		
Variable	LN-positive<br>n = 55 (45.5%)	LN-negative<br>n = 66 (54.5%)	*p*-value
Age	33.6±13.0	38.3±13.1	<0.01
Gender			
Female	48 (87.3%)	62 (93.9%)	0.63
Male	7 (12.7%)	4 (6.1%)	
Clinical manifestations			
Fever	15 (27.3%)	18 (27.3%)	1
Cutaneous manifestations	35 (63.6%)	24 (36.4%)	< 0.01
Joint manifestations	18 (32.7%)	39 (59.1%)	< 0.01
Serositis	7 (12.7%)	13 (19.7%)	0.03
Edema	21 (38.2%)	2 (3.0%)	< 0.01
Hematological manifestations	12 (21.8%)	19 (28.8%)	0.38
Neuropsychiatric involvement	2 (3.6%)	7 (10.6%)	0.15
Digestive involvement	10 (18.2%)	6 (9.1%)	0.14
Titers of ANA			< 0.01
1:100	4 (7.3%)	7 (10.6%)	
1:320	10 (18.2%)	21 (31.8%)	
1:1000	19 (34.5%)	30 (45.5%)	
1:3200	22(40.0%)	8 (12.1%)	
Patterns of ANA			0.01
Homogeneous/speckled (AC-1 /AC4-5)	26 (47.3%)	24 (36.4%)	
Speckled (AC4-5)	10 (18.2%)	29 (43.9%)	
Homogeneous (AC-1)	12 (21.8%)	4 (6.1%)	
Speckled/cytoplasmic speckled (AC4-5/AC18-20)	5 (9.1%)	6 (9.1%)	
Nucleolar (AC8-10)	2 (3.6%)	3 (4.5%)	
Extractable Nuclear Antigen (ENA)			
Anti-U1-nRNP/Sm	28 (50.9%)	37 (56.1%)	0.57
Anti-Sm	17 (30.9%)	11 (16.7%)	0.06
Anti-Ro-52	29 (52.7%)	39 (59.1%)	0.48
Anti-SSA	32 (58.2%)	38 (57.6%)	0.96
Anti-SSB	7 (12.3%)	7 (10.6%)	0.72
AnuA	18 (32.7%)	16 (24.2%)	0.3
AHA	12 (21.8%)	11 (16.7%)	0.47
Anti-P	17 (30.9%)	15 (22.7%)	0.31
Renal biopsy histological classification			
II	4 (7.27%)		
III	6 (10.91%)		
IV	20 (36.36%)		
V	7 (12.73%)		
III+IV	6 (10.91%)		
III+V	9 (16.36%)		
IV+V	3 (5.45%)		

Supplementary Figures

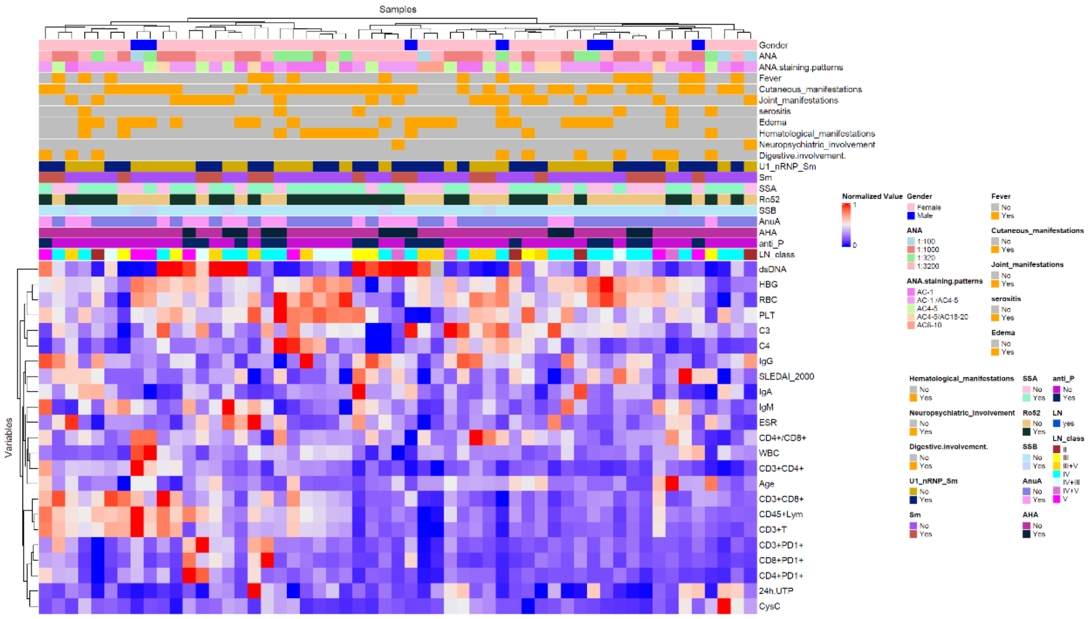

Figure S1. Heatmap of demographic, clinical, biochemical, immunological, and histological features in LN patients. The quantitative variables were normalized (centered) across samples.
